# A Fast HPLC/UV Method for Determination of Ketoprofen in Cellular Media

**DOI:** 10.1002/open.202300147

**Published:** 2023-11-13

**Authors:** Oleksandra Vozniuk, Zdeněk Kejík, Kateřina Veselá, Markéta Skaličková, Petr Novotný, Róbert Hromádka, Jan Hajduch, Pavel Martásek, Milan Jakubek

**Affiliations:** ^1^ Department of Paediatrics and Inherited Metabolic Disorders First Faculty of Medicine Charles University and General University Hospital in Prague Ke Karlovu 455/2 128 08 Prague Czech Republic; ^2^ BIOCEV First Faculty of Medicine Charles University 252 20 Vestec Czech Republic; ^3^ Department of Analytical Chemistry Faculty of Chemical Engineering University of Chemistry and Technology 166 28 Prague Czech Republic

**Keywords:** HPLC/UV, ketoprofen, cellular medium

## Abstract

A simple, sensitive and quick HPLC method was developed for the determination of ketoprofen in cell culture media (EMEM, DMEM, RPMI). Separation was performed using a gradient on the C18 column with a mobile phase of acetonitrile and miliQ water acidified by 0.1 % (v/v) formic acid. The method was validated for parameters including linearity, accuracy, precision, limit of quantitation and limit of detection, as well as robustness. The response was found linear over the range of 3–100 μg/mL as demonstrated by the acquired value of correlation coefficient R2=0.9997. The described method is applicable for determination of various pharmacokinetic aspects of ketoprofen *in vitro*.

## Graphical abstract

A simple, sensitive and quick HPLC method was developed for the determination of ketoprofen in cell culture media. Separation was performed using a gradient on a C18 column with a mobile phase of acetonitrile and acidified miliQ water. The method was validated for linearity, accuracy, precision, limit of quantitation, limit of detection, as well as for robustness. The described method is applicable for determination of various pharmacokinetic aspects of ketoprofen *in vitro*.

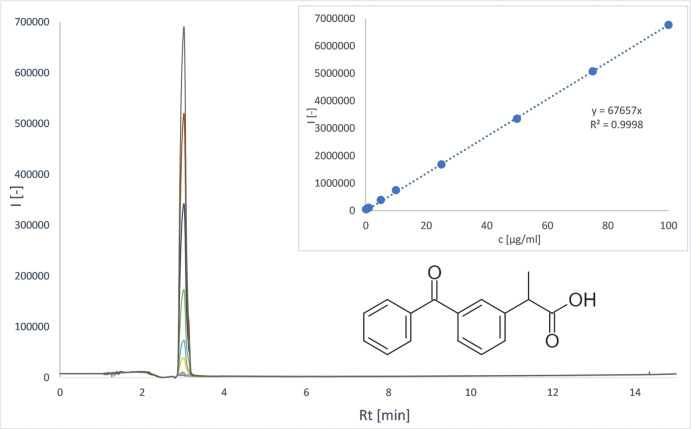

## Introduction

Non‐steroidal anti‐inflammatory drugs (NSAIDs) are one of the most used pharmaceuticals due to their ability to suppress acute and chronic pain without developing tolerance or addiction. Anti‐inflammatory, analgesic and antipyretic effects of NSAIDs are mediated by their ability to stop the biosynthesis of prostaglandins from arachidonic acid by inhibiting enzyme cyclooxygenase (COX). COX exists in two isoforms: the constitutively expressed COX‐1 isoform and the inducible COX‐2 isoform, which induces inflammation and the feeling of pain.[^1^, ^2^]

Ketoprofen (2‐(3‐benzoylphenyl)propionic acid) is an effective nonsteroidal anti‐inflammatory and analgesic drug, which belongs to the group of substituted 2‐phenylpropionic acids. It is often prescribed for the treatment of traumatic and postoperative pain, pain caused by inflammation, and arthritis. It can be administered in different formulations: tablets, capsules, injectable solutions, topical gels, and ointments. There are different dosages of ketoprofen in oral tablets or capsules: most common are 50, 75, 100, 150 and 200 mg. The choice of the dosage depends on the type of pain, age, weight of the patient and on the number of administrations during day. Adverse drug effects are similar as for other NSAIDs: nausea, epigastric discomfort, indigestion, and in some cases gastrointestinal ulceration or bleeding.[^3^, ^4^]

**Figure 1:**
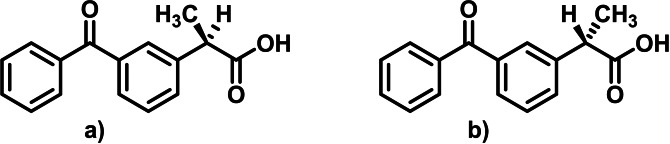
Structure of **(a)** R(−)‐ketoprofen; **(b)** S(+)‐ketoprofen.

Properties of ketoprofen such as octanol‐water partition coefficient log P=2.8 and acid dissociation constant p*K*_a_=3.7^[5]^ indicate that this drug is acidic, but slightly hydrophobic. Ketoprofen has chiral center and occurs as S(+)‐ and R(−)‐enantiomer (Figure 1), but only S(+)‐ketoprofen is pharmacologically active. Despite that, ketoprofen is administered as racemic mixture because, in an organism, it can undergo chiral inversion.^[6]^

After administration the drug is quickly absorbed and then in the bloodstream it is 99 % bound to plasma proteins, mostly to albumin. Peak plasma concentration of ketoprofen differs from administered dose: for 50 mg it is 3.2–4.8 μg/mL, for 100 mg it ranges around 5.5–10.1 μg/mL, and for 150 mg it is 13.1 μg/mL. Metabolism of ketoprofen involves one major pathway – conjugation with glucuronic acid, which leads to formation of an unstable glucuronic ester that is excreted in the urine. Other possible metabolites are formed from ketoprofen by hydroxylation of aromatic ring or by reduction of the ketone group.[^1^, ^2^] Different pharmacokinetic aspects (absorption,^[3]^ release,^[4]^ bioavailability,^[7]^ stability^[8]^ of the drug) are often studied and evaluated using *in vitro* tests. Therefore, it is desirable to develop determination method for solutions of the drug in various cultivation media, since cell cultures for *in vitro* tests are usually maintained in DMEM (Dulbecco's Modified Eagle Medium) or other media like EMEM (Eagle's minimum essential medium) or RPMI (Roswell Park Memorial Institute).[^3^, ^9^]

Determination of ketoprofen concentration in biological fluids as is blood serum, plasma or urine has been described and carried out by different techniques: gas chromatography,^[10]^ capillary electrophoresis,^[11]^ UV spectrophotometry^[12]^ and high‐performance liquid chromatography (HPLC).[^13^, ^14^, ^15^, ^16^] Gas chromatography is not suited for quick routine analysis since it requires long sample preparation before analysis. Capillary electrophoresis is one of the cheapest and easiest methods, but it is limited by low sensitivity and amount of the sample (more detailed comparison is in Table S1). Therefore, HPLC is the most used technique for determination of ketoprofen. A lot of HPLC methods are focused on the separation of individual enantiomers, that requires either derivatisation of analyte or use of special columns or additions of chiral compounds to mobile phase. Because ketoprofen is usually administered as racemate and the chiral inversion process occurs in the organism, it is enough to determine total amount of ketoprofen, which simplifies the analysis.

No HPLC method for effective *in vitro* determination of ketoprofen in media has been described so far. Therefore, in this study, we have developed and validated a simple method for the determination of ketoprofen concentration by measuring media samples containing ketoprofen.

## Materials and Methods

### Chemicals and Reagents

Ketoprofen (purity min. 99.5 %, Ph.Eur.), Zentiva, (Czech Republic). Methanol (ROTISOLV® >99.98 %, Ultra LC–MS) and acetonitrile (ROTISOLV® HPLC), Carl Roth (Germany), formic acid (HCOOH) (LC–MS grade, LiChropurTM), Sigma‐Aldrich (USA). For HPLC analysis was used miliQ water. For method verification were used different medias: EMEM, ATCC (USA), DMEM, BIOSERA (France), RPMI, Thermo Fisher Scientific (USA).

### Instrumentation and chromatographic conditions

HPLC system LCMS 2020, Shimadzu (Japan) was used for chromatographic separation. Separation was performed gradient on Shim‐pack GIST C18 column (5 μm, Shimadzu) with acetonitrile and miliQ water acidified by 0.1 % (v/v) formic acid as the mobile phase at flow rate of 1 mL/min. Injection volume was 50 μL, the analysis lasted 15 min and was carried out at temperature 40±1 °C. An UV detector operating at wavelengths of 200–600 nm was used for signal recording, and chromatograms were recorded at λ=254 nm.

### Preparation of standard solutions

Ketoprofen stock solution in methanol (200 μg/mL) was prepared by dissolving 0.01 g of ketoprofen in 50 mL of methanol. Nine calibration solutions of 0.1, 0.5, 1, 5, 10, 25, 50, 75 and 100 μg/mL were prepared by diluting the stock solution with the determined volume of methanol.

Ketoprofen stock solutions in cell culture media (200 μg/mL) were prepared by dissolving 0.01 g of the drug in 50 mL of the selected medium (EMEM, DMEM, RPMI). Seven calibration solutions of 1, 5, 10, 25, 50, 75 and 100 μg/mL were prepared by diluting the stock solution with the determined volume of tested medium.

### Sample preparation

1 mL of the stock solution of ketoprofen in medium were taken and extracted five times in an extraction funnel with 1 mL of diethyl ether. The obtained 5 mL of the organic phase were evaporated in a 10 mL flask on a rotary vacuum evaporator at a pressure of 666 Torr in a water bath with temperature of 40 °C. After evaporation of the solvent, the pressure was gradually reduced to about 3 Torr for complete drying. The dry residue was redissolved in 1.5 mL of mobile phase, then 1 mL of this solution was taken for analysis by HPLC. This procedure was applied to all three stock solutions and repeated three times.

### HPLC method validation

Developed method was validated by following parameters:

#### Linearity and range

These properties were tested on ketoprofen solutions with concentrations of 0.1, 0.5, 1, 5, 10, 25, 50, 75 and 100 μg/mL. After measuring all calibration solutions, blank was subtracted from their chromatograms. Microsoft Excel was used to plot the linearity graph and get the linear regression equation:

(1) y=a·x,

where *y* is the response of the detector *x* is the analytical concentration, coefficients *a* and *b* represent the sensitivity of the analysis and the intercept respectively.

The square of correlation coefficient *R* ^2^ has to be greater than 0.995 in order to consider chosen concentration range linear.^[17]^

#### Limit of detection and limit of quantification

LOD and LOQ were determined by equations (2) and (3) using data obtained from linearity testing.

(2) $$LOD=\;\frac{\left|a\right|+3\cdot S_{a}}{b},$$

(3) $$LOQ=\;\frac{\left|a\right|+10\cdot S_{a}}{b},$$

where *a* and *b* are coefficients from equation (1) and *S*_a_ and *S*_b_ are their standard deviations.^[17]^

In order to calculate standard deviations of the coefficients, following parameters were calculated:

(4) $$\sum xx=\sum {\left(X-\bar{X}\right)}^{2}=\sum X^{2}-{\left(\sum X\right)}^{2}$$

(5) $$\sum xy=\sum XY-\left(\sum X\right)\left(\sum Y\right)/n$$

(6) $$\sum yy=\sum {\left(Y-\bar{Y}\right)}^{2}=\sum Y^{2}-{\left(\sum Y\right)}^{2}$$

where *X* and *Y* are the variables, $\bar{X}\;a\;\bar{Y\;}$ are their means and *n* is the number of measurements.

Parameters obtained from equations (4), (5) and (6) are used to calculate the standard deviation of the individual deviations of measured values in *Y*, above and below the linear line:

(7) $$S_{y,x}=\sqrt{\frac{\sum yy-{\left(\sum xy\right)}^{2}/\sum xx}{n-2}}$$

The standard deviations for coefficients *a* and *b* are calculated by using *S*_*y.x*_, from equation 7:

(8) $$S_{a}=S_{y.x}\sqrt{\frac{\sum X^{2}}{n\sum xx}}$$

(9) $$S_{b}=S_{y.x}\sqrt{\frac{1}{n\sum xx}}$$

#### Precision

Ketoprofen solutions with concentration of 2.4, 50 and 80 μg/mL were independently measured six times by using developed method in order to evaluate the precision and capability of the HPLC system. For the evaluation of the method precision stock solutions of ketoprofen in different media were measured multiple times including the sample preparation process (extraction, evaporation and redissolution). The relative standard deviation RSD must not be higher than 2 %.^[17]^

#### Accuracy

Three concentration levels of ketoprofen solutions (5, 50 and 80 μg/mL) were measured three times under the same conditions as calibration solutions. Linear regression equation (1) obtained from the measurement results of the calibration solutions was used to calculate amount of ketoprofen in tested solutions. Accuracy was calculated as:

(10) $$Accuracy\;\left(\%\right)=\;\frac{Measured\;concentration\;of\;ketoprofen}{Theoretical\;concentration\;of\;ketoprofen}\cdot 100\;\%$$

For the pharmaceutical industry the acceptance criterium for accuracy of the determination of API (active pharmaceutical ingredient) concentration is 100±2 %. Lower percent recoveries may also be acceptable depending on the needs of the methods.^[18]^

#### Robustness

As part of the robustness testing were varied following internal factors of the analytical method:

- flow rate was changed from 1 mL/min to 0.5 and 2 mL/min
- composition of the mobile phase was modified by using isocratic elution of acetonitrile and acetonitrile with addition formic acid to final concentration 1 %.

## Results and Discussion

### Optimization of HPLC conditions

**Figure 2:**
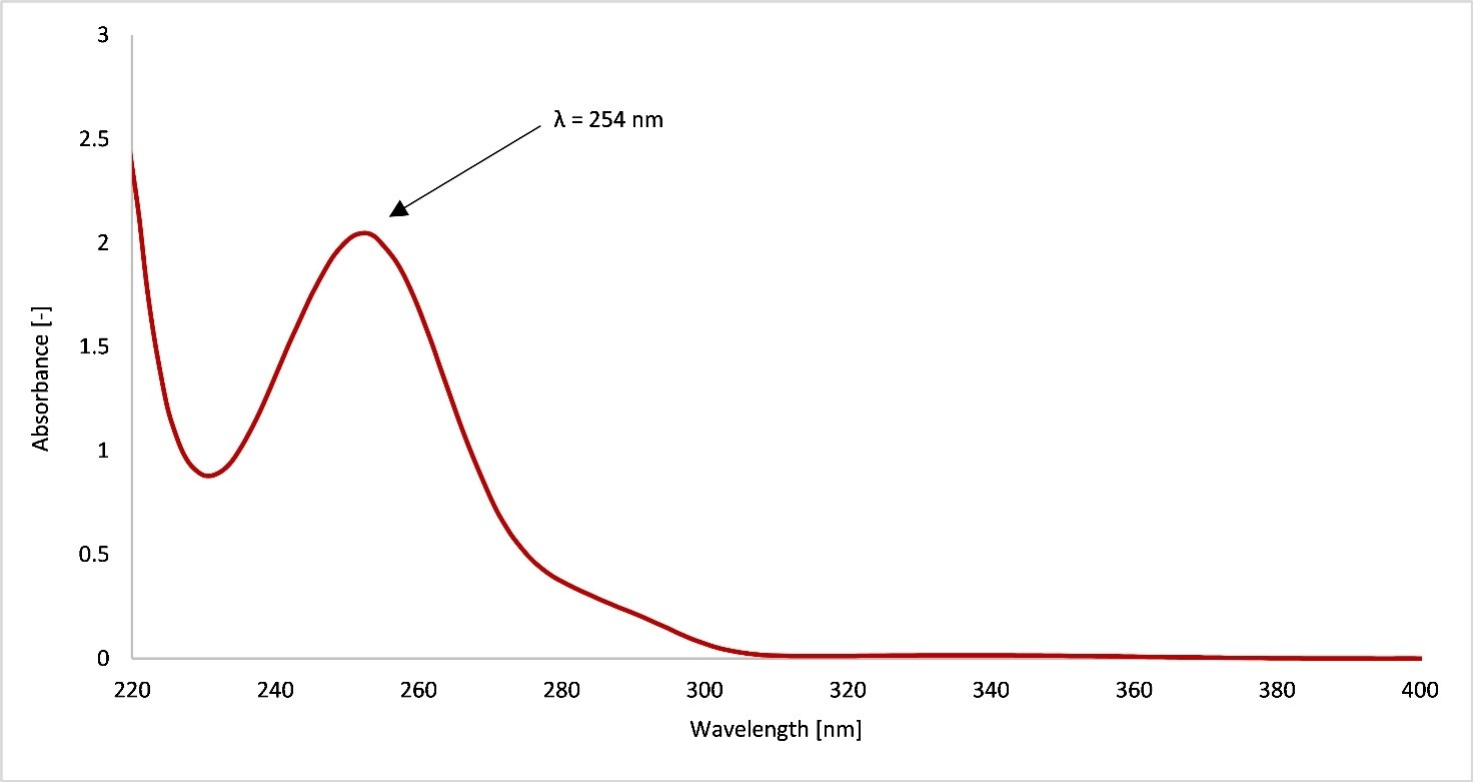
UV absorption spectrum of ketoprofen.

Since ketoprofen is a polar substance, the separation was carried out on a non‐polar C18 stationary phase. Based on the literature were tried out various mobile phases with different composition, polarity and acidity. The best results for the separation of ketoprofen and good resolution of peaks were obtained using acetonitrile acidified with 0.1 % (v/v) of formic acid. For this mobile phase was chosen flowrate 1 mL/min since it enabled to get quite sharp and well resolved peak. The temperature on the column was 40±1 °C, which is the standard temperature for separations on the used HPLC system. Chromatograms were recorded at wavelength *λ*=254 nm, as this is the absorption maximum of ketoprofen (Figure 2) and concentration of ketoprofen was equal 50 μg/mL. Retention time for ketoprofen was 3.06 min, but the analysis time was kept for 15 min in order to completely elute any other compounds (for example, from the media) and prepare the column for the next separation. Analysis time can be shortened up to 5 min for quick determination of the drug in routine analysis.

### Method validation

#### Linearity and range

**Figure 3:**
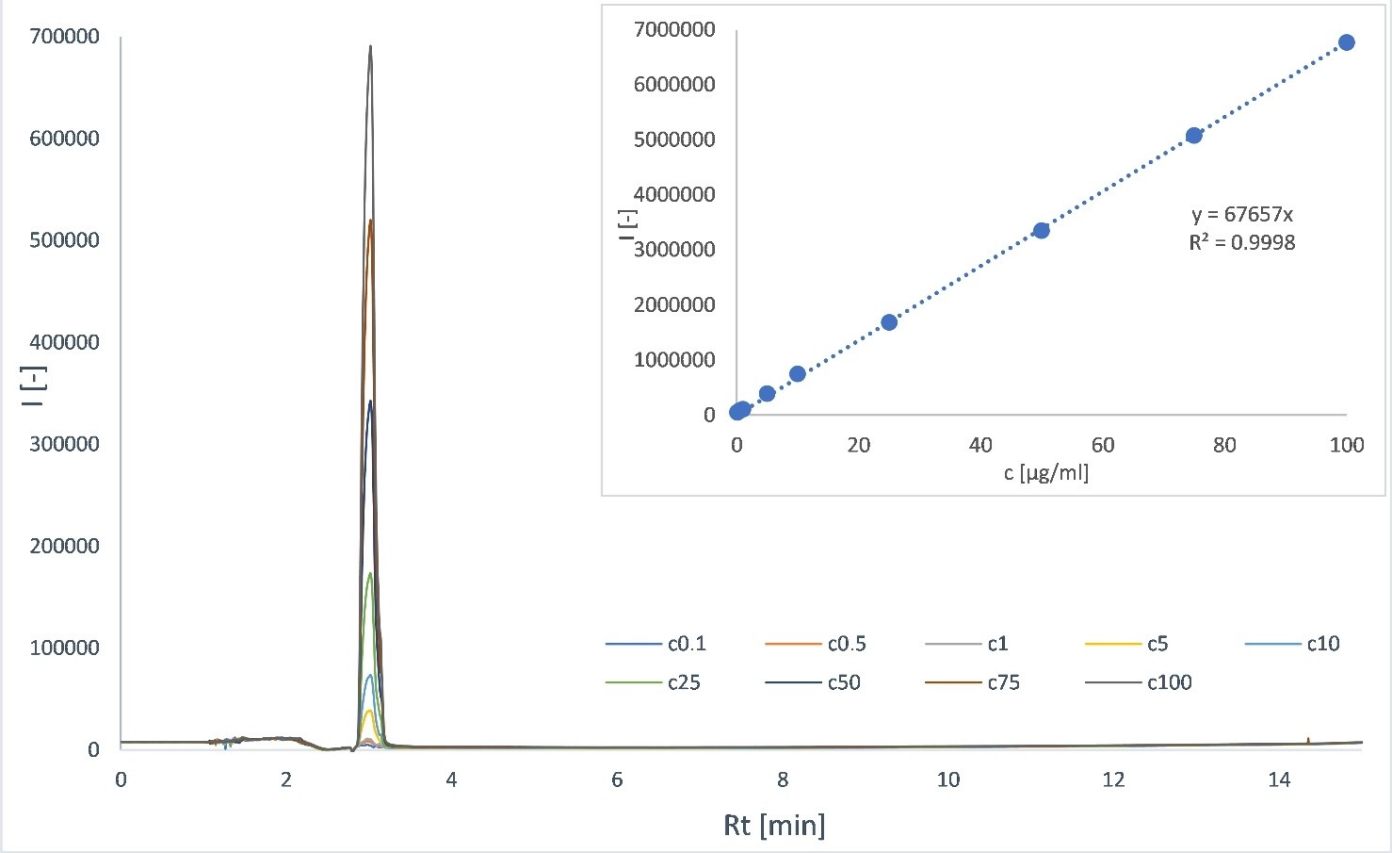
Overlay of ketoprofen calibration curve chromatograms and calibration curve.

After measuring all calibration solutions, blank was subtracted from their chromatograms. Peak area was plotted against concentration of ketoprofen in the range of 0.1–100 μg/mL (Figure 3). The data were analysed using linear regression and coefficients *a* and *b* from equation (1) were obtained. The value of square of correlation coefficient *R* ^2^ was determined as 0.9997 that is higher than 0.995, which means that detector response is linear in chosen concentration range.

#### Limit of detection and limit of quantification

Using the least squares method and equations (2) and (3), the limit of detection value was determined as LOD=1.20 μg/mL and the limit of quantification value was determined as LOQ=2.43 μg/mL. It means that the linear range must be adjusted to 3–100 μg/mL, as concentrations 0.1, 0.5 and 1 μg/mL cannot be reliably detected and determined using this method.

#### Precision

In order to calculate the relative standard deviation, mean and standard deviation (SD) were calculated from peak areas from six independent measurements of ketoprofen solutions on three concentration levels (Table 1). Its value for ketoprofen solution with concentration of 2.4 μg/mL was determined as RSD=0.47 % and as RSD=0.11 % for 50 and 80 μg/mL solutions. The RSD for 2.4 μg/mL ketoprofen solution is higher than for other two solutions, which can be related to the fact, that this concentration is the limit of quantification, where measurements are a little less precise. All values are less than 2 %, which proves the capability of the HPLC system on different concentration levels.

**Table 1:** Data for evaluation of the precision of the HPLC system.

	C_ketoprofen_=2.4 μg/mL		C_ketoprofen_=50 μg/mL		C_ketoprofen_=80 μg/mL	
N^o^	Retention time (min)	Peak area	Retention time (min)	Peak area	Retention time (min)	Peak area
1	3.043	221747	3.062	3559005	3.033	5434368
2	3.044	222946	3.062	3555515	3.033	5444309
3	3.044	223778	3.062	3561641	3.033	5448011
4	3.047	222605	3.060	3567539	3.036	5446462
5	3.058	224343	3.061	3561733	3.036	5451912
6	3.045	224340	3.062	3560105	3.037	5444489
	**Mean**	223293	**Mean**	3560923	**Mean**	5444925
	**SD**	1040	**SD**	3963	**SD**	5877
	**RSD (%)**	0.47	**RSD (%)**	0.11	**RSD (%)**	0.11

The same way was calculated relative standard deviations of the repeated determination of ketoprofen concentration in different media (Table 2 and extended version of the table in the Supplementary Table S2). Smaller value of RSD for the determination in EMEM compared to the determinations in DMEM or RPMI can be caused by simpler composition of this medium. All RSD values are less than 2 %, that confirms the precision of the developed method.

**Table 2:** Data for evaluation of the precision of the developed method.

Medium type	Mean	SD	RSD (%)
EMEM	9865229	28865	0.29
DMEM	7211350	87233	1.21
RPMI	11492191	10493	0.09

#### Accuracy

In order to evaluate accuracy of the developed method, nine measurements were carried out on three concentration levels. Concentrations of the ketoprofen in tested samples were calculated using the linear regression equation (y=67657⋅x) and then compared to the injected concentrations (Table 3). Accuracies for ketoprofen solutions with concentration of 50 and 80 μg/mL fall within the acceptance range from 98 to 102 %. Accuracy for the 5 μg/mL ketoprofen solution is a little higher, but is still acceptable, considering that this concentration is close to the limit of the quantification.

**Table 3:** Data for evaluation of the accuracy of the method.

Injected concentration of ketoprofen (μg/mL)	Average peak area of ketoprofen	Determined concentration of ketoprofen (μg/mL)	Accuracy (%)
5	355740	5.258	105.16
50	3423213	50.597	101.19
80	5428734	80.239	100.30

#### Robustness

**Figure 4:**
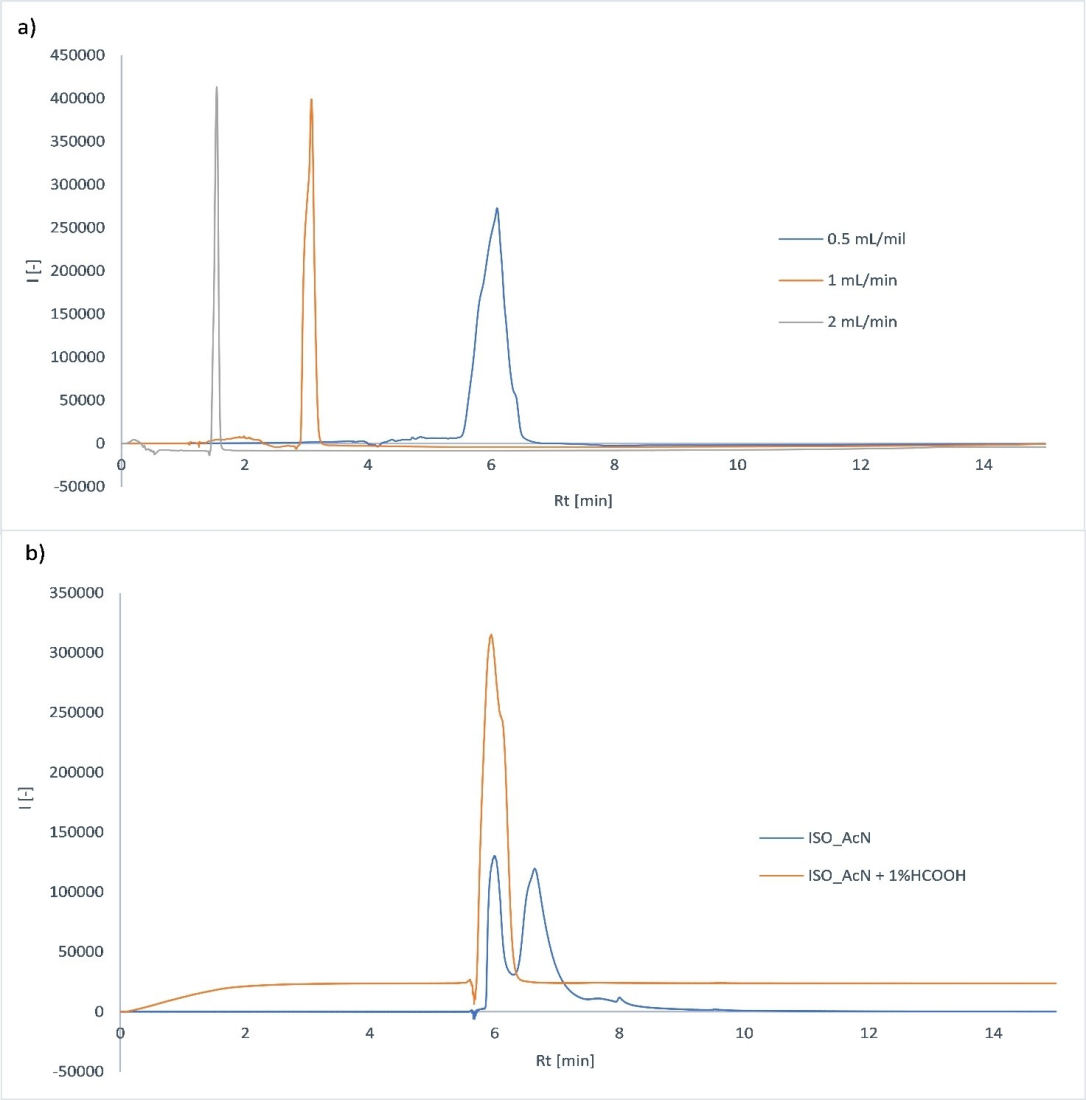
Chromatograms depicting the effect on the separation of ketoprofen (50 μg/mL) after the change in **(a)** flow rate; **(b)** Isocratic elution.

1. Effect of changing mobile phase flow rate (Figure 4, a)

Reducing the flow rate to 0.5 mL/min led to broadening of the peak, more pronounced tailing and a significant increase in the retention time of ketoprofen. When the flow rate was increased to 2 mL/min, the retention time of ketoprofen decreased and the peak slightly narrowed.

2. Effect of changing the composition of the mobile phase (Figure 4, b)

Using isocratic elution of acetonitrile with HCOOH addition led to broadening of the peak, more pronounced tailing and a significant increase in the retention time of ketoprofen. Using isocratic elution of non‐acidified acetonitrile leads to a significant broadening and splitting of the peak – this mobile phase is unsuitable for analysis.

### Method application for the determination of ketoprofen in media

#### Determination of ketoprofen after previous extraction

Extraction of 1 mL of ketoprofen solution (0.2 mg) in medium was carried out using diethyl ether. For each type of medium, the extraction was performed three times (Table 4).

**Table 4:** Measurement results of ketoprofen solutions after extraction from media.

Medium type	Average Rt of ketoprofen (min)	Average peak area of ketoprofen	Average Rt of medium (min)	Average peak area of medium	The result after reading the media	Recovery (%)
EMEM	3.118	9865229	3.331	2207327	7657902	85.29±0.64
DMEM	3.100	7211350	3.331	401612	6809738	75.9±1.9
RPMI	3.117	11492191	3.187	2505830	8986361	100.09±0.23

**Figure 5:**
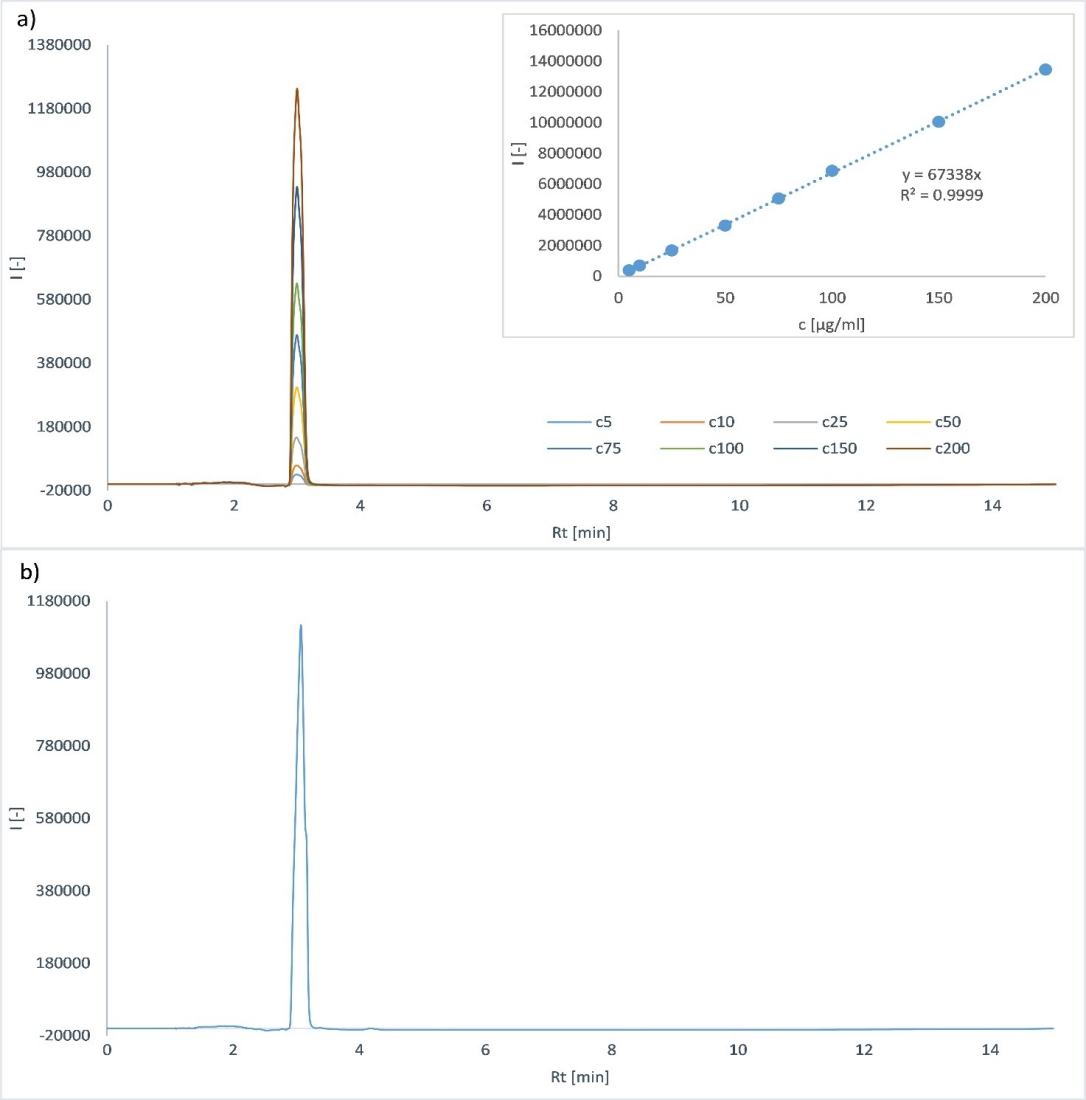
**(a)** Overlay of ketoprofen calibration curve chromatograms and calibration curve; **(b)** Example of the chromatogram after the extraction; used medium was DMEM; for extraction was used the highest concentration level of ketoprofen 200 μg/mL.

After the organic phase was evaporated and the dry residue was redissolved in 1.5 mL of the mobile phase, the chromatograms of the obtained ketoprofen solutions were measured using the developed method. In order to determine the amount of ketoprofen after extraction, calibration solutions were also measured under the same conditions (Figure 5).

Linear regression equation (y=67338 ⋅ x) was obtained from the measurement results of the calibration solutions. It was used to calculate the concentration of ketoprofen in solution after the extraction. Subsequently, the weight of ketoprofen was calculated.

The recovery (Table 4) was calculated as the ratio of the determined weight of ketoprofen after extraction to the known amount of ketoprofen in the stock solution (around 0.2 mg) before extraction. The results are listed with the 95 % confidence interval calculated from standard deviation with coefficient *k*=2.

## Discussion

In this study was developed and validated method enabling determination of ketoprofen in the calibration range of 5–100 μg/mL. The method was developed in this concentration range, because IC_50_ (concentration, which leads to half the maximum inhibitory effect on the action of pro‐inflammatory factor) for S‐ketoprofen is 26.7 μg/ml^[19]^ and even higher concentrations of racemic ketoprofen are used for *in vitro* studies.[^20^, ^21^, ^22^] For example, in the study by Hara‐Yamamura et al. HepG2 cells gene expression changes started increasing around 127 μg/mL ketoprofen exposure.^[22]^ In the study by Banach et al. the same concentration of ketoprofen in combination with UVA radiation reduced melanoma cells vitality.^[21]^ Chosen concentration range is also applicable for pharmacokinetic and bioavailability studies, since total bioavailability of ketoprofen is dose proportional in the range of 50–200 mg,^[2]^ so its concentration in blood ranges respectively from 10 to 40 μg/mL (considering the blood volume of an adult about 5 L) and differs from patient to patient.[^15^, ^23^] Developed method is precise, gives a linear response in the chosen range. Compared to the other methods of determination of ketoprofen (Table S1), this method is linear in the similar range, doesn't require almost any sample or mobile phase preparation, which makes it easy and efficient for quick routine analysis.[^10^, ^11^, ^24^] The advantage of the developed method over method described by Zafar et al. is that we used a binary gradient in our method, while their method uses isocratic elution. Usage of gradient leads to decreased consumption of organic solvent. In addition, it is well known that a proper choice of gradient elution can optimize the separation of multicomponent mixtures, which is mostly the case for real samples.^[23]^

The robustness of the method to different changes in experiment conditions was thoroughly tested. Small changes in column temperature do not significantly affect the separation efficiency. Changing flow rate to 2 mL/min had positive effect on the separation, but it was not very significant, and therefore using 1 mL/min flow rate has more advantages, as there is smaller usage of the mobile phase and the peak area is bigger, so lower concentrations can be detected. Method is also sufficiently robust to changes in the composition of mobile phase as long as its pH stays slightly acidic.

The developed method was applied to ketoprofen solutions in three different media. While located in the bloodstream, 99 % of ketoprofen is bound to blood proteins, mainly to albumin.^[2]^ It binds to the Sudlow II site of albumin, which contains the amino acids leucine, isoleucine, alanine, asparagine, phenylalanine, glycine, cysteine, tyrosine, arginine, lysine and serine.^[25]^ Nevertheless, in this case of free amino acids, interaction of ketoprofen could be expected for the tryptophan, valine and leucine.[^26^, ^27^] The used media (EMEM, DMEM, RPMI) contain these amino acids (Table S3), so it can be assumed that ketoprofen dissolved in media also occurred there mainly in bound form. When calibration solutions of ketoprofen in media were measured (Figure S1), it was visible, that ketoprofen peak was deformed, which proved the assumption that media have some impact on ketoprofen. Due to the peak shape the determination of the peak area wouldn't be precise and calculation of linear regression equation was impossible. In according above, the lowest recovery was observed in DMEM, which contains most of these amino acid (Table S3). Therefore, it wasn't possible to determine ketoprofen concentration directly in the media and the extraction was needed.

In order to separate unwanted components, extraction into diethyl ether was performed, followed by analysis using the developed HPLC method. The amount of ketoprofen after extraction was determined by the calibration curve method. The extraction yield varied between 75–100 % depending on the type of the used medium. The recovery values could be affected by ability of ketoprofen to bind to proteins found in the medium. In addition, the recovery varies depending on the type of medium that was used, which may be a consequence of their different composition (Table S3). DMEM has twice the amount of amino acids compared to EMEM, which may have led to a higher degree of ketoprofen binding and consequently a lower recovery after the extraction. Compared to EMEM, DMEM and RPMI contain more types of amino acids that are also present in the binding site of albumin for ketoprofen, which may also cause the difference in recovery values. We attribute this difference to the matrix effect, which is correlated with the effect of the components present in the medium, i. e. the presence of different amino acids in the medium. We assume that in the case of medium with a different composition, this matrix effect would be significantly lower.

## Conclusions

All of the set goals for this study have been met. The developed method is precise, gives a linear response in the chosen range and is sufficiently robust. This method is potentially applicable for the quick routine determination of ketoprofen in *in vitro* model, which is important, for example, for designing pharmacokinetic studies. In the future, the method will be optimized in order to improve the detection limit and increase the extraction yield.

## Supporting Information Summary

The Supporting Information contains:

Figure S1: Overlay of ketoprofen calibration curve chromatograms measured in used media; Table S1: Comparison of different methods for the determination of ketoprofen;

Table S2. Detailed table of data for evaluation of the precision of the developed method;

Table S3. Comparison of the composition of used media.

## Author Contributions

**O. Vozniuk, K. Veselá, M. Skaličková**, **P. Novotný, R. Hromádka**, **J. Hajduch**: Investigation, Methodology, Validation, Writing – original draft. **Z. Kejík**: Conceptualization, Investigation, Writing – original draft; **P. Martásek**: Conceptualization, Resources, Supervision. **M. Jakubek**: Conceptualization, Resources, Visualization, Supervision, Writing – original draft. The publication has been approved by all of authors.

## Conflict of interests

The authors declare no conflict of interest. The funders had no role in the design of the study; in the collection, analyses, or interpretation of data; in the writing of the manuscript; or in the decision to publishHConflictOfInterest the results.

1

## Supporting information

As a service to our authors and readers, this journal provides supporting information supplied by the authors. Such materials are peer reviewed and may be re‐organized for online delivery, but are not copy‐edited or typeset. Technical support issues arising from supporting information (other than missing files) should be addressed to the authors.

Supporting Information

## Data Availability

The data that support the findings of this study are available in the supplementary material of this article.
