# Supplementary material for: A Fast HPLC/UV Method for Determination of Ketoprofen in Cellular Media
Source: ChemistryOpen. 2023 Nov 13;13(3):e202300147. doi: 10.1002/open.202300147 (PMC10924040; doi:10.1002/open.202300147)
Supplement: Supplementary file 1 — Supporting Information [file OPEN-13-e202300147-s001.pdf]

ChemistryOpen

Supporting Information

A Fast HPLC/UV Method for Determination of Ketoprofen in Cellular Media

Oleksandra Vozniuk, Zdeněk Kejík, Kateřina Veselá, Markéta Skaličková, Petr Novotný, Róbert Hromádka, Jan Hajduch, Pavel Martásek, and Milan Jakubek*

Context:

Figure S1. Overlay of ketoprofen calibration curve chromatograms measured in used media.
Table S1. Comparison of different methods for the determination of ketoprofen.
Table S2. Detailed table of data for evaluation of the precision of the developed method.
Table S3. Comparison of the composition of used media.

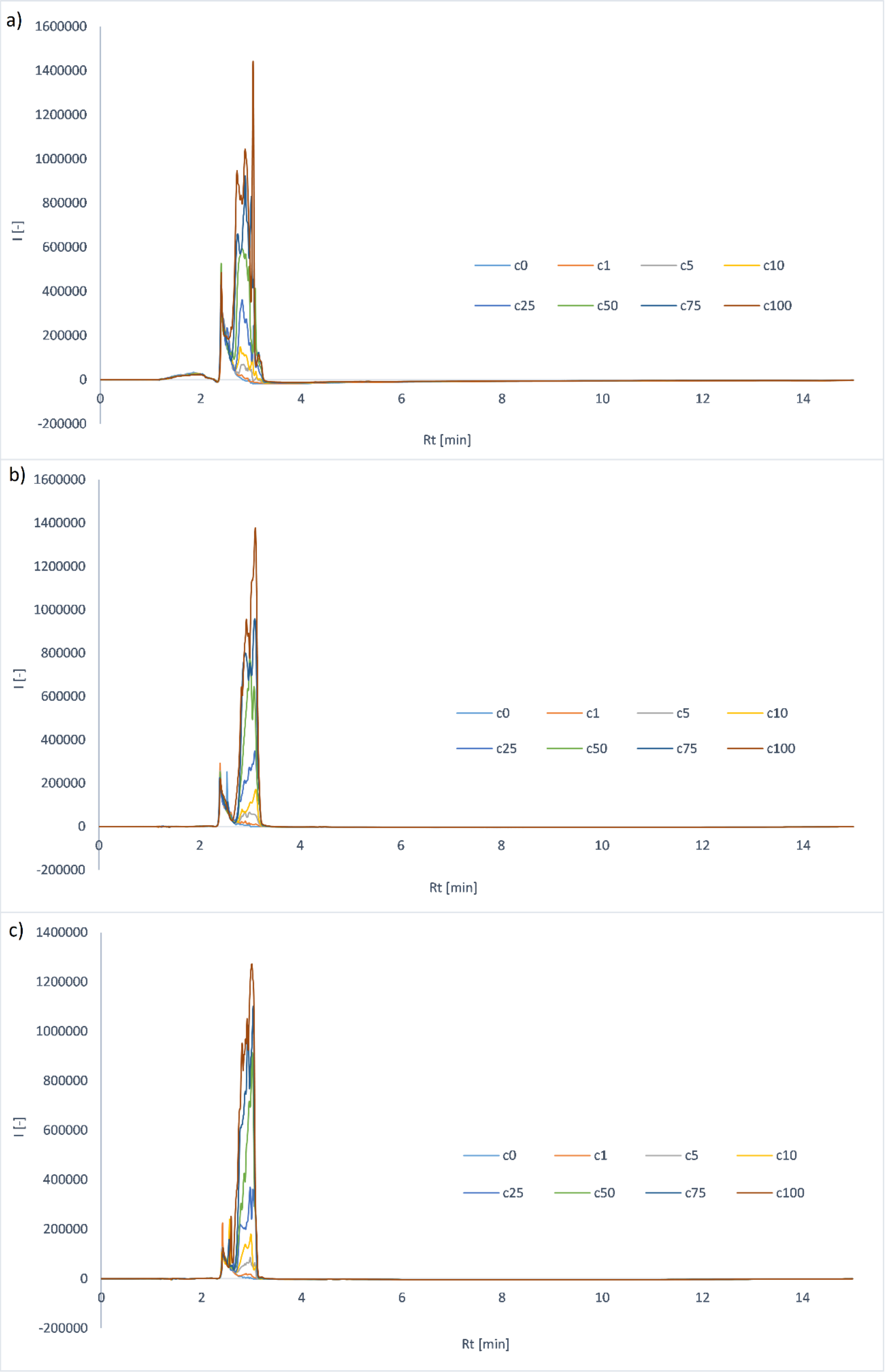

Figure S1. Overlay of ketoprofen calibration curve chromatograms measured in **(a)** DMEM, **(b)** EMEM, **(c)** RPMI

Table S1. Comparison of different methods for the determination of ketoprofen

Name of the method	Sample preparation	Conduction of the experiment	Application
Gas chromatography [1]	100 μL of 2-(4-benzoylphenyl)butanoic acid (internal standard), 0.4 mL of 0.2 M tetrabutylammonium hydroxide solution and 3 mL of 1.1 M iodomethane solution in CH_2Cl_2 were added to 400 μL of plasma containing ketoprofen. The mixture was stirred for 70 min to allow methylation to occur. Subsequently, the organic layer was evaporated and the dry residue was dissolved in 1 mL of toluene.	The separation took place on a glass column filled with 3% OV-17. The temperature of the injector was set to 250 °C, in the thermostat to 225 °C. Nitrogen was used as carrier gas with a flow rate of 30 ml/min. The injection was 1-3 μL of the sample. The ^{63}Ni electron capture detector was heated to 240 °C to achieve the lowest possible detection limit.	This method is suitable for the determination of ketoprofen in plasma down to 0.5 μM, but requires time-consuming extractive methylation of ketoprofen and the internal standard.
Capillary electrophoresis [2]	200 μL of acetonitrile containing 10 mg/L isobutylmethylxanthine (internal standard) was added to 100 μL of serum containing ketoprofen. After mixing and centrifugation, the supernatant was collected for further analysis.	The fused silica capillary was filled with a separation buffer: 0.25 M boric acid and 2 M sodium hydroxide (pH 8.9), with addition of acetonitrile and β-cyclodextrin. The device was set at 12 kV, 30 °C, the sample was injected hydrodynamically (99 s with a pressure of 3447.4 Pa). The current in the beginning of the analysis was 35 μA and was increased to 65 μA after the exclusion of acetonitrile, the analysis lasted 12 min. The spectrophotometric absorption UV detector was set at 254 nm.	A simple and rapid method for the determination of ketoprofen in the linear range of 1 to 10 mg/L. The LOD was 0.6 mg/L and LOQ was 1 mg/L. This method is cheaper than HPLC analysis, but it is less sensitive and more affected by the matrix.

High performance liquid chromatography [3]	Plasma containing ketoprofen was mixed with acetonitrile in a ratio of 1:1 in order to separate the protein. This mixture was vortexed for 10 min, centrifuged at 3000 rpm for 10 min. Supernatant was then filtered and then used to produce the desired concentrations of standard solutions.	The separation was carried out isocratically on a Discovery HS C18 column, 5 µm (25 cm × 4.6 mm). The mobile phase was a mixture of methanol: water (70:30 v/v) with pH = 3.3 (adjusted with phosphoric acid). Sample injection was 20 µl, flow rate was 1 mL/min. The diode array detector was set at 260 nm.	This method illustrated linearity from 0.2441 to 125 µg/mL, with LOD = 0.122 µg/mL and LOQ = 0.2441 µg/mL. It is an easy, quick and cost effective method that can be used in pharmacokinetic studies of ketoprofen.
Developed method	Stock solutions of ketoprofen in media extracted three times with diethyl ether in a ratio of 1:1. The obtained organic phase was evaporated, the dry residue was redissolved in 3 mL of mobile phase, then 1 mL of this solution was taken for analysis by HPLC	Separation was performed gradient on Shim-pack GIST C18 column (5 µm, Shimadzu) with acetonitrile and miliQ water acidified by 0.1% (v/v) formic acid as the mobile phase at flow rate of 1 mL/min. Injection volume was 50 µL, the analysis lasted 15 min. The diode array detector was set at 254 nm.	Developed method demonstrated linearity in the range 3 - 100 µg/mL. LOD was determined as 1.20 µg/mL and LOQ was determined as 2.43 µg/mL. It is suited for routine determination of ketoprofen concentration in cultivation media.

Table S2. Detailed table of data for evaluation of the precision of the developed method

	EMEM		DMEM		RPMI	
**№**	**Retention time (min)**	**Peak area (mV.s)**	**Retention time (min)**	**Peak area (mV.s)**	**Retention time (min)**	**Peak area (mV.s)**
1	3.118	9833278	3.108	7110719	3.033	3.118
2	3.117	9872987	3.095	7257837	3.033	3.117
3	3.118	9889423	3.098	7265494	3.033	3.117
	**Mean (mV.s)**	9865229.33	**Mean (mV.s)**	7211350	**Mean (mV.s)**	11492191
	**SD (mV.s)**	28865	**SD (mV.s)**	87233	**SD (mV.s)**	10493
	**RSD (%)**	0.29	**RSD (%)**	1.21	**RSD (%)**	0.09

Table S3. Comparison of the composition of used media

Compounds	Concentration (mg/L)		
	EMEM	DMEM	RPMI
Glycine	0	30	10
L-Arginine	0	0	200
L-Arginine hydrochloride	126	84	0
L-Asparagine	0	0	50
L-Aspartic acid	0	0	20
L-Cystine-2HCl	31	63	65
L-Glutamic acid	0	0	20
L-Glutamine	292	0	300
L-Histidine	0	0	15
L-Histidine hydrochloride-H_2O	42	42	0
L-Hydroxyproline	0	0	20
L-Isoleucine	52	105	50
L-Leucine	52	105	50
L-Lysine hydrochloride	73	146	40
L-Methionine	15	30	15
L-Phenylalanine	32	66	15
L-Proline	0	0	20
L-Serine	0	42	30
L-Threonine	48	95	20
L-Tryptophan	10	16	5
L-Tyrosine disodium salt dihydrate	52	104	29
L-Valine	46	94	20
Biotin	0	0	0.2
Choline chloride	1	4	3
D-Calcium pantothenate	1	4	0.25
Folic acid	1	4	1
i-Inositol	2	7.2	35
Niacinamide	1	4	1
Para-aminobenzoic acid	0	0	1
Pyridoxal hydrochloride	1	0	0
Pyridoxine hydrochloride	0	4	1
Riboflavin	0.1	0.4	0.2
Thiamine hydrochloride	1	4	1
Vitamin B12	0	0	0.005
Calcium chloride ($CaCl_2$) (anhyd.)	200	200	0
Calcium nitrate ($Ca(NO_3)_2$-$4H_2O$)	0	0	100
Ferric nitrate ($Fe(NO_3)_3$-$9H_2O$)	0	0.1	0
Magnesium sulfate ($MgSO_4$) (anhyd.)	97.67	97.67	48.84
Potassium chloride (KCl)	400	400	400
Sodium bicarbonate ($NaHCO_3$)	2200	3700	2000
Sodium chloride (NaCl)	6800	6400	6000
Sodium phosphate dibasic (Na_2HPO_4) (anhyd.)	0	0	800

Sodium phosphate monobasic (NaH_2PO_4-H_2O)	140	125	
D-Glucose (dextrose)	1000	1000	2000
Glutathione (reduced)	0	0	1
Phenol red	10	10	0
Sodium pyruvate	0	110	0

References:

1. P. Stenberg, T. E. Jönsson, B. Nilsson, F. Wollheim, *J. Chromatogr.* 1979, **177**, 145-148.
2. M. Friedberg, Z. K. Shihabi, *J. Chromatogr. B Biomed. Appl.* 1997, **695**, 193-198.
3. F. Zafar, M. H. Shoaib, A. Naz, R. I. Yousuf, H. Ali, *Am. J. Anal. Chem.* 2013, **5**, 6.
